# Tri­phenyl­telluronium(IV) bromide acetone hemisolvate

**DOI:** 10.1107/S160053681302816X

**Published:** 2013-10-19

**Authors:** Sari M. Närhi, Raija Oilunkaniemi, Risto S. Laitinen

**Affiliations:** a Department of Chemistry, PO Box 3000, FI-90014 University of Oulu, Finland

## Abstract

The asymmetric unit of the title compound, 2C_18_H_15_Te^+^·2Br^−^·C_3_H_6_O or Ph_3_TeBr·0.5Me_2_CO, contains two crystallographically independent tri­phenyl­telluronium cations, two bromide anions, and one disordered [site-occupancy ratio = 0.581 (7):0.419 (7)] solvent mol­ecule. Inter­ionic Te⋯Br inter­actions connect the cations and anions into a tetra­meric step-like structure. The primary coordination spheres of both Te atoms are TeC_3_ trigonal pyramids: three short secondary tellurium–bromine inter­actions expand the coordination geometry of one of the Te atoms to an octa­hedron. While the other Te atom shows only two Te⋯Br secondary bonding inter­actions, it is also six-coordinated due to a Te⋯π inter­action [3.769 (2) Å] with one of the phenyl rings of the adjacent cation.

## Related literature

For the structures of unsolvated tri­phenyl­telluronium chloride and Ph_3_TeCl^.^ 0.5CHCl_3_, see: Ziolo & Extine (1980 ▶) and Collins *et al.* (1988 ▶), respectively. For the preparation of [(Ph_3_PO)_2_H]_2_[Te_2_Br_10_], see: Närhi *et al.* (2004 ▶). For Te⋯π inter­actions, see: Zukerman-Schpector & Haiduc (2002 ▶). For Te—C bond lengths in tri­phenyl­telluronium cations, see: Oilunkaniemi *et al.* (2001 ▶).
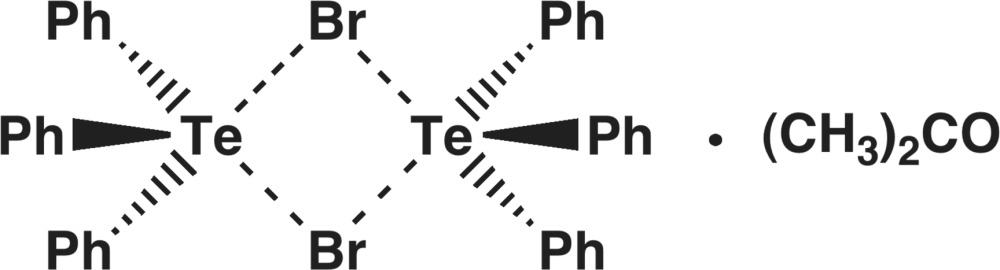


## Experimental

### 

#### Crystal data

2C_18_H_15_Te^+^·2Br^−^·C_3_H_6_O
*M* *_r_* = 935.70
Triclinic, 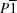

*a* = 11.753 (2) Å
*b* = 13.086 (3) Å
*c* = 13.165 (3) Å
α = 77.69 (3)°
β = 66.05 (3)°
γ = 81.73 (3)°
*V* = 1804.16 (2) Å^3^
*Z* = 2
Mo *K*α radiation
μ = 3.86 mm^−1^
*T* = 150 K
0.30 × 0.28 × 0.25 mm

#### Data collection

Bruker–Nonius KappaCCD diffractometer
Absorption correction: multi-scan (*XPREP* in *SHELXTL*; Sheldrick, 2008 ▶) *T*_min_ = 0.327, *T*_max_ = 0.381
28482 measured reflections
7083 independent reflections
6013 reflections with *I* > 2σ(*I*)
*R*_int_ = 0.065

#### Refinement

*R*[*F* ^2^ > 2σ(*F* ^2^)] = 0.037
*wR*(*F* ^2^) = 0.106
*S* = 1.01
7083 reflections
415 parameters
9 restraints
H-atom parameters constrained
Δρ_max_ = 1.18 e Å^−3^
Δρ_min_ = −1.86 e Å^−3^

### 

Data collection: *COLLECT* (Bruker, 2008 ▶); cell refinement: *DENZO-SMN* (Otwinowski & Minor, 1997 ▶); data reduction: *DENZO-SMN*; program(s) used to solve structure: *SHELXS97* (Sheldrick, 2008 ▶); program(s) used to refine structure: *SHELXL97* (Sheldrick, 2008 ▶); molecular graphics: *DIAMOND* (Brandenburg, 2006 ▶); software used to prepare material for publication: *WinGX* (Farrugia, 2012 ▶).

## Supplementary Material

Crystal structure: contains datablock(s) I. DOI: 10.1107/S160053681302816X/hb7149sup1.cif

Structure factors: contains datablock(s) I. DOI: 10.1107/S160053681302816X/hb7149Isup2.hkl

Supplementary material file. DOI: 10.1107/S160053681302816X/hb7149Isup3.cml

Additional supplementary materials:  crystallographic information; 3D view; checkCIF report

## Figures and Tables

**Table 1:** Selected bond lengths (Å)

Te1—C121	2.135 (4)
Te1—C111	2.150 (4)
Te1—C131	2.156 (4)
Te1—Br1	3.4481 (9)
Te1—Br1^i^	3.3941 (9)
Te1—Br2	3.4174 (10)
Te2—C211	2.120 (4)
Te2—C231	2.134 (4)
Te2—C221	2.148 (4)
Te2—Br1^i^	3.3922 (9)
Te2—Br2	3.3527 (9)

Symmetry code: (i) 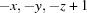.

**Fig. 1.**
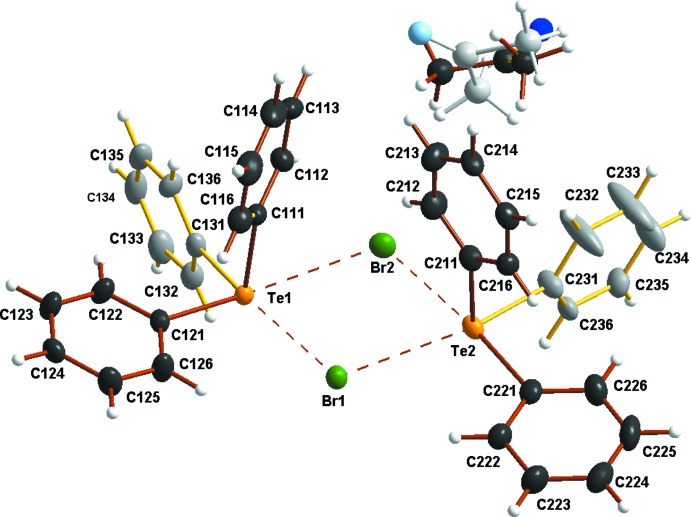
The asymmetric unit of triphenyltellurium bromide indicating the interionic Te···Br contacts. The thermal ellipsoids have been drawn at 50% probability.

**Fig. 2.**
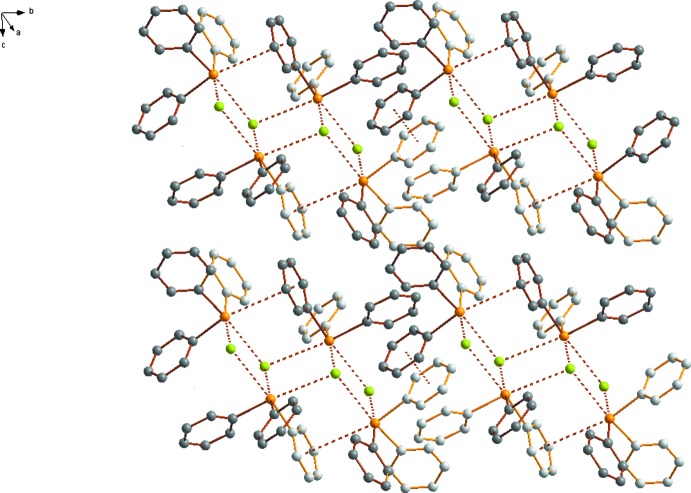
The two-dimensional planar packing of the tetrameric (Ph3TeBr)4 units. The solvent molecules and the hydrogen atoms have been omitted for clarity.

## supplementary crystallographic information

### 1. Comment

The asymmetric unit in the title compound contains two crystallographically independent triphenyltelluronium cations, two bromide anions, and one disordered acetone molecule (see Fig. 1). The solvent molecule can be located in two alternative positions with the site occupation factors 58 (1):42 (1).

The Te-C bond lengths in Ph_3_TeBr^.^ 1/2 Me_2_CO range from 2.120 (4) to 2.156 (4) Å, which are normal for triphenyltelluronium cations (see for example Oilunkaniemi *et al.*, 2001.) The primary coordination geometry of tellurium atoms can be depicted as a pyramidal AX_3_E environment (X = bonding pair and E = lone pair) . The C-Te-C bond angles range from 91.36 (14) to 96.04 (15)°.

The structural features of organotellurium salts R_3_TeX are governed by weak tellurium-anion secondary bonding interactions, which expand the AX_3_E trigonal pyramidal geometry around the tellurium atom generally into a six-coordinate environment. The two crystallographically independent tellurium atoms in the title compound, however, show different coordination environments. The trigonal pyramidal geometry around the Te1 atom is expanded into an octahedron by three Te···Br contacts, whereas Te2 shows two Te···Br contacts. Te2 becomes six-coordinated through the Te···π interaction with one of the phenyl rings of an adjacent cation. These interactions expand the coordination of the tellurium atoms into AX_3_Y_3_E and AX_3_Y_2_ZE environments, respectively. The Te···Br distances range from 3.3529 (5) Å to 3.4483 (4) Å.

The interionic Te···Br contacts build a tetrameric step-like unit in the lattice. This kind of polymeric structures are common in tellurium-halogen compounds, especially with heavier halogens. Ph_3_TeCl^.^ 1/2 CHCl_3_, for example, shows a similar tetrameric structure as the title compound (Collins *et al.*, 1988). In fact, Ph_3_TeBr^.^ 1/2 Me_2_CO is isomorphic with Ph_3_TeCl^.^ 1/2 CHCl_3_. The unsolvated Ph_3_TeCl consists of dimeric structural units (Ziolo *et al.*, 1980).

The (Ph_3_TeBr)_4_ tetramers form two-dimensional network in the crystal as shown in Fig. 2. The planes are linked together by the solvent molecules.

### 2. Experimental

A few colorless crystals of Ph_3_TeBr^.^ 1/2 Me_2_CO were obtained by slow evaporation of the solvent from the acetone solution of the precipitate isolated from the reaction of [(Ph_3_PO)_2_H]_2_[Te_2_Br_10_] (94.5 mg; 0.04 mmol) and Ph_3_TeCl (56.3 mg; 0.14 mmol) in CH_2_Cl_2_.

### 3. Refinement

The solvent molecule was found to be disordered and refined in two positions. Since the site occupation factors and thermal parameters of the disordered atoms correlate with each other, the thermal parameters of the corresponding pairs of atoms were restrained to be equal. The site occupation factors were 58 (1):42 (1) after the final refinement.

H atoms were positioned geometrically and refined using a riding model with 0.98 Å and *U*_iso_(H) = 1.5 *U*_eq_(C) and 0.95 Å and *U*_iso_(H) = 1.2 *U*_eq_(C) for the methyl and aromatic H atoms, respectively.

### Crystal data

**Table:**

2C_18_H_15_Te^+^·2Br^−^·C_3_H_6_O	*Z* = 2
*M**_r_* = 935.70	*F*(000) = 904
Triclinic, *P*1	*D*_x_ = 1.722 Mg m^−^^3^
Hall symbol: -P 1	Mo *K*α radiation, λ = 0.71073 Å
*a* = 11.753 (2) Å	Cell parameters from 6013 reflections
*b* = 13.086 (3) Å	θ = 1.6–26.0°
*c* = 13.165 (3) Å	µ = 3.86 mm^−^^1^
α = 77.69 (3)°	*T* = 150 K
β = 66.05 (3)°	Block, colorless
γ = 81.73 (3)°	0.30 × 0.28 × 0.25 mm
*V* = 1804.16 (2) Å^3^	

### Data collection

**Table:**

Bruker–Nonius KappaCCD diffractometer	7083 independent reflections
Radiation source: fine-focus sealed tube	6013 reflections with *I* > 2σ(*I*)
Graphite monochromator	*R*_int_ = 0.065
φ scans, and ω scans with κ offsets	θ_max_ = 26.0°, θ_min_ = 2.4°
Absorption correction: multi-scan (*XPREP* in *SHELXTL*; Sheldrick, 2008)	*h* = −14→14
*T*_min_ = 0.327, *T*_max_ = 0.381	*k* = −16→16
28482 measured reflections	*l* = −16→16

### Refinement

**Table:**

Refinement on *F*^2^	Secondary atom site location: difference Fourier map
Least-squares matrix: full	Hydrogen site location: inferred from neighbouring sites
*R*[*F*^2^ > 2σ(*F*^2^)] = 0.037	H-atom parameters constrained
*wR*(*F*^2^) = 0.106	*w* = 1/[σ^2^(*F*_o_^2^) + (0.0676*P*)^2^ + 1.4955*P*] where *P* = (*F*_o_^2^ + 2*F*_c_^2^)/3
*S* = 1.01	(Δ/σ)_max_ = 0.001
7083 reflections	Δρ_max_ = 1.18 e Å^−^^3^
415 parameters	Δρ_min_ = −1.86 e Å^−^^3^
9 restraints	Extinction correction: *SHELXL97* (Sheldrick, 2008), Fc^*^=kFc[1+0.001xFc^2^λ^3^/sin(2θ)]^-1/4^
Primary atom site location: structure-invariant direct methods	Extinction coefficient: 0.0030 (3)

### Special details

**Table:**

Geometry. All e.s.d.'s (except the e.s.d. in the dihedral angle between two l.s. planes) are estimated using the full covariance matrix. The cell e.s.d.'s are taken into account individually in the estimation of e.s.d.'s in distances, angles and torsion angles; correlations between e.s.d.'s in cell parameters are only used when they are defined by crystal symmetry. An approximate (isotropic) treatment of cell e.s.d.'s is used for estimating e.s.d.'s involving l.s. planes.
Refinement. Refinement of *F*^2^ against ALL reflections. The weighted *R*-factor *wR* and goodness of fit *S* are based on *F*^2^, conventional *R*-factors *R* are based on *F*, with *F* set to zero for negative *F*^2^. The threshold expression of *F*^2^ > σ(*F*^2^) is used only for calculating *R*-factors(gt) *etc*. and is not relevant to the choice of reflections for refinement. *R*-factors based on *F*^2^ are statistically about twice as large as those based on *F*, and *R*- factors based on ALL data will be even larger.

### Fractional atomic coordinates and isotropic or equivalent isotropic displacement parameters (Å^2^)

**Table:**

	*x*	*y*	*z*	*U*_iso_*/*U*_eq_	Occ. (<1)
Te1	0.12350 (2)	0.076353 (18)	0.298305 (18)	0.01941 (10)	
Te2	0.22042 (2)	0.211171 (18)	0.57690 (2)	0.02422 (10)	
Br1	0.03931 (3)	−0.16756 (3)	0.45590 (3)	0.02334 (11)	
Br2	0.38302 (4)	0.12124 (3)	0.33426 (3)	0.02978 (12)	
C111	0.1352 (4)	0.2366 (3)	0.2135 (3)	0.0255 (8)	
C112	0.2503 (4)	0.2762 (3)	0.1487 (3)	0.0316 (9)	
H112	0.3247	0.2330	0.1403	0.038*	
C113	0.2556 (5)	0.3806 (4)	0.0958 (4)	0.0398 (11)	
H113	0.3342	0.4089	0.0504	0.048*	
C114	0.1469 (5)	0.4432 (4)	0.1092 (4)	0.0449 (12)	
H114	0.1510	0.5144	0.0729	0.054*	
C115	0.0334 (5)	0.4031 (3)	0.1747 (4)	0.0425 (12)	
H115	−0.0408	0.4468	0.1837	0.051*	
C116	0.0255 (4)	0.2993 (3)	0.2279 (4)	0.0322 (9)	
H116	−0.0533	0.2716	0.2734	0.039*	
C121	−0.0417 (4)	0.0626 (3)	0.2732 (3)	0.0233 (8)	
C122	−0.0377 (4)	0.0475 (4)	0.1704 (3)	0.0327 (9)	
H122	0.0404	0.0372	0.1107	0.039*	
C123	−0.1463 (4)	0.0473 (4)	0.1552 (4)	0.0368 (10)	
H123	−0.1426	0.0367	0.0847	0.044*	
C124	−0.2618 (4)	0.0626 (3)	0.2412 (3)	0.0288 (9)	
H124	−0.3368	0.0649	0.2294	0.035*	
C125	−0.2649 (4)	0.0742 (4)	0.3435 (4)	0.0380 (10)	
H125	−0.3431	0.0828	0.4036	0.046*	
C126	−0.1564 (4)	0.0735 (4)	0.3609 (3)	0.0348 (10)	
H126	−0.1604	0.0805	0.4326	0.042*	
C131	0.2471 (3)	0.0094 (3)	0.1525 (3)	0.0251 (8)	
C132	0.2916 (4)	−0.0921 (3)	0.1739 (4)	0.0307 (9)	
H132	0.2685	−0.1285	0.2493	0.037*	
C133	0.3715 (4)	−0.1418 (4)	0.0836 (4)	0.0403 (11)	
H133	0.4018	−0.2126	0.0970	0.048*	
C134	0.4056 (4)	−0.0863 (4)	−0.0255 (4)	0.0414 (11)	
H134	0.4600	−0.1193	−0.0871	0.050*	
C135	0.3618 (4)	0.0155 (4)	−0.0453 (4)	0.0387 (11)	
H135	0.3868	0.0525	−0.1205	0.046*	
C136	0.2809 (4)	0.0655 (3)	0.0435 (3)	0.0317 (9)	
H136	0.2497	0.1359	0.0298	0.038*	
C211	0.1911 (4)	0.3664 (3)	0.4988 (3)	0.0261 (8)	
C212	0.2340 (4)	0.3877 (3)	0.3824 (4)	0.0358 (10)	
H212	0.2698	0.3327	0.3398	0.043*	
C213	0.2242 (5)	0.4900 (3)	0.3283 (4)	0.0392 (10)	
H213	0.2540	0.5055	0.2483	0.047*	
C214	0.1706 (4)	0.5694 (3)	0.3915 (4)	0.0308 (9)	
H214	0.1649	0.6395	0.3546	0.037*	
C215	0.1261 (4)	0.5474 (3)	0.5069 (4)	0.0298 (9)	
H215	0.0875	0.6021	0.5494	0.036*	
C216	0.1368 (4)	0.4461 (3)	0.5622 (3)	0.0260 (8)	
H216	0.1075	0.4312	0.6421	0.031*	
C221	0.1197 (4)	0.2423 (3)	0.7456 (3)	0.0297 (9)	
C222	−0.0063 (4)	0.2247 (3)	0.7947 (3)	0.0353 (10)	
H222	−0.0449	0.2006	0.7540	0.042*	
C223	−0.0756 (5)	0.2426 (4)	0.9041 (4)	0.0448 (12)	
H223	−0.1619	0.2304	0.9388	0.054*	
C224	−0.0189 (5)	0.2782 (4)	0.9624 (4)	0.0458 (12)	
H224	−0.0666	0.2912	1.0369	0.055*	
C225	0.1064 (5)	0.2948 (4)	0.9130 (4)	0.0492 (13)	
H225	0.1447	0.3194	0.9536	0.059*	
C226	0.1774 (5)	0.2761 (4)	0.8043 (4)	0.0413 (11)	
H226	0.2643	0.2863	0.7708	0.050*	
C231	0.3977 (4)	0.2389 (3)	0.5710 (4)	0.0317 (9)	
C232	0.4446 (6)	0.3359 (4)	0.5336 (8)	0.084 (3)	
H232	0.3977	0.3942	0.5097	0.100*	
C233	0.5605 (6)	0.3485 (4)	0.5308 (8)	0.094 (3)	
H233	0.5931	0.4158	0.5044	0.113*	
C234	0.6299 (5)	0.2645 (4)	0.5659 (5)	0.0522 (14)	
H234	0.7087	0.2741	0.5653	0.063*	
C235	0.5833 (4)	0.1677 (3)	0.6012 (4)	0.0330 (9)	
H235	0.6302	0.1094	0.6251	0.040*	
C236	0.4676 (4)	0.1540 (3)	0.6025 (3)	0.0268 (8)	
H236	0.4368	0.0861	0.6251	0.032*	
O1A	0.7532 (15)	0.4177 (18)	0.1670 (15)	0.164 (5)	0.419 (7)
C10A	0.6491 (16)	0.4044 (18)	0.1894 (16)	0.100 (4)	0.419 (7)
C11A	0.599 (3)	0.346 (2)	0.139 (2)	0.160 (10)	0.419 (7)
H11A	0.6470	0.3553	0.0573	0.240*	0.419 (7)
H11B	0.5114	0.3702	0.1537	0.240*	0.419 (7)
H11C	0.6028	0.2711	0.1713	0.240*	0.419 (7)
C12A	0.557 (3)	0.500 (2)	0.236 (3)	0.153 (8)	0.419 (7)
H12A	0.6006	0.5445	0.2582	0.230*	0.419 (7)
H12B	0.4844	0.4734	0.3020	0.230*	0.419 (7)
H12C	0.5305	0.5408	0.1774	0.230*	0.419 (7)
O1B	0.5289 (12)	0.4528 (13)	0.0876 (11)	0.164 (5)	0.581 (7)
C10B	0.5732 (13)	0.4293 (14)	0.1552 (12)	0.100 (4)	0.581 (7)
C11B	0.623 (2)	0.3333 (13)	0.1992 (18)	0.153 (8)	0.581 (7)
H11D	0.6129	0.3348	0.2764	0.230*	0.581 (7)
H11E	0.7114	0.3231	0.1520	0.230*	0.581 (7)
H11F	0.5779	0.2754	0.1995	0.230*	0.581 (7)
C12B	0.581 (3)	0.5240 (15)	0.2095 (17)	0.160 (10)	0.581 (7)
H12D	0.6611	0.5177	0.2174	0.240*	0.581 (7)
H12E	0.5129	0.5223	0.2840	0.240*	0.581 (7)
H12F	0.5734	0.5904	0.1608	0.240*	0.581 (7)

### Atomic displacement parameters (Å^2^)

**Table:**

	*U*^11^	*U*^22^	*U*^33^	*U*^12^	*U*^13^	*U*^23^
Te1	0.01891 (15)	0.02137 (15)	0.01801 (14)	−0.00127 (10)	−0.00629 (10)	−0.00527 (10)
Te2	0.02140 (15)	0.02096 (15)	0.03317 (17)	−0.00101 (10)	−0.01357 (11)	−0.00474 (11)
Br1	0.0229 (2)	0.0232 (2)	0.0252 (2)	0.00093 (15)	−0.00996 (16)	−0.00700 (15)
Br2	0.0245 (2)	0.0294 (2)	0.0321 (2)	0.00201 (17)	−0.00752 (17)	−0.00790 (17)
C111	0.032 (2)	0.0247 (19)	0.0221 (19)	−0.0065 (16)	−0.0130 (16)	−0.0016 (15)
C112	0.035 (2)	0.031 (2)	0.032 (2)	−0.0088 (18)	−0.0149 (18)	−0.0036 (17)
C113	0.054 (3)	0.041 (3)	0.025 (2)	−0.021 (2)	−0.015 (2)	0.0044 (18)
C114	0.076 (4)	0.025 (2)	0.043 (3)	−0.013 (2)	−0.033 (3)	0.0006 (19)
C115	0.062 (3)	0.026 (2)	0.051 (3)	0.010 (2)	−0.035 (3)	−0.012 (2)
C116	0.039 (2)	0.028 (2)	0.033 (2)	0.0007 (18)	−0.0160 (18)	−0.0083 (17)
C121	0.0256 (19)	0.0218 (18)	0.0267 (19)	−0.0017 (15)	−0.0138 (16)	−0.0047 (15)
C122	0.028 (2)	0.050 (3)	0.021 (2)	−0.0019 (19)	−0.0088 (16)	−0.0084 (18)
C123	0.033 (2)	0.057 (3)	0.025 (2)	−0.006 (2)	−0.0149 (18)	−0.0086 (19)
C124	0.022 (2)	0.033 (2)	0.034 (2)	−0.0059 (17)	−0.0140 (17)	−0.0035 (17)
C125	0.025 (2)	0.055 (3)	0.034 (2)	−0.008 (2)	−0.0052 (18)	−0.018 (2)
C126	0.025 (2)	0.055 (3)	0.026 (2)	−0.0037 (19)	−0.0056 (16)	−0.0193 (19)
C131	0.0185 (18)	0.033 (2)	0.025 (2)	−0.0012 (16)	−0.0063 (15)	−0.0127 (16)
C132	0.023 (2)	0.039 (2)	0.031 (2)	0.0002 (17)	−0.0084 (16)	−0.0149 (18)
C133	0.030 (2)	0.048 (3)	0.048 (3)	0.006 (2)	−0.014 (2)	−0.026 (2)
C134	0.027 (2)	0.067 (3)	0.036 (3)	−0.001 (2)	−0.0072 (19)	−0.031 (2)
C135	0.029 (2)	0.065 (3)	0.021 (2)	−0.014 (2)	−0.0027 (17)	−0.014 (2)
C136	0.034 (2)	0.040 (2)	0.0195 (19)	−0.0042 (19)	−0.0061 (16)	−0.0096 (17)
C211	0.0235 (19)	0.0221 (19)	0.036 (2)	0.0034 (15)	−0.0163 (17)	−0.0054 (16)
C212	0.046 (3)	0.028 (2)	0.035 (2)	0.0027 (19)	−0.016 (2)	−0.0123 (18)
C213	0.058 (3)	0.031 (2)	0.032 (2)	0.001 (2)	−0.021 (2)	−0.0077 (18)
C214	0.039 (2)	0.0182 (19)	0.042 (2)	−0.0027 (17)	−0.0245 (19)	−0.0016 (17)
C215	0.029 (2)	0.0237 (19)	0.040 (2)	0.0008 (16)	−0.0140 (18)	−0.0115 (17)
C216	0.027 (2)	0.026 (2)	0.0251 (19)	−0.0020 (16)	−0.0083 (16)	−0.0065 (16)
C221	0.038 (2)	0.027 (2)	0.029 (2)	−0.0027 (17)	−0.0199 (18)	−0.0009 (16)
C222	0.041 (2)	0.040 (2)	0.028 (2)	−0.007 (2)	−0.0149 (19)	−0.0056 (18)
C223	0.051 (3)	0.055 (3)	0.029 (2)	−0.010 (2)	−0.012 (2)	−0.010 (2)
C224	0.067 (3)	0.044 (3)	0.033 (2)	−0.004 (2)	−0.025 (2)	−0.008 (2)
C225	0.072 (4)	0.048 (3)	0.049 (3)	−0.009 (3)	−0.041 (3)	−0.013 (2)
C226	0.044 (3)	0.044 (3)	0.047 (3)	−0.008 (2)	−0.026 (2)	−0.010 (2)
C231	0.025 (2)	0.025 (2)	0.049 (3)	0.0005 (16)	−0.0196 (19)	−0.0043 (18)
C232	0.049 (3)	0.028 (3)	0.192 (8)	−0.009 (2)	−0.077 (4)	0.009 (4)
C233	0.061 (4)	0.030 (3)	0.214 (9)	−0.014 (3)	−0.085 (5)	0.007 (4)
C234	0.035 (3)	0.039 (3)	0.097 (4)	−0.002 (2)	−0.040 (3)	−0.012 (3)
C235	0.035 (2)	0.033 (2)	0.040 (2)	0.0024 (18)	−0.0224 (19)	−0.0094 (18)
C236	0.0228 (19)	0.030 (2)	0.032 (2)	−0.0074 (16)	−0.0129 (16)	−0.0047 (16)
O1A	0.119 (8)	0.242 (13)	0.118 (8)	−0.103 (9)	−0.048 (7)	0.061 (9)
C10A	0.067 (8)	0.143 (12)	0.070 (8)	−0.058 (9)	−0.012 (5)	0.030 (8)
C11A	0.23 (2)	0.148 (15)	0.072 (9)	−0.145 (16)	−0.007 (11)	0.018 (9)
C12A	0.112 (12)	0.099 (12)	0.18 (2)	−0.042 (10)	0.026 (12)	−0.031 (13)
O1B	0.119 (8)	0.242 (13)	0.118 (8)	−0.103 (9)	−0.048 (7)	0.061 (9)
C10B	0.067 (8)	0.143 (12)	0.070 (8)	−0.058 (9)	−0.012 (5)	0.030 (8)
C11B	0.112 (12)	0.099 (12)	0.18 (2)	−0.042 (10)	0.026 (12)	−0.031 (13)
C12B	0.23 (2)	0.148 (15)	0.072 (9)	−0.145 (16)	−0.007 (11)	0.018 (9)

### Geometric parameters (Å, º)

**Table:**

Te1—C121	2.135 (4)	C213—C214	1.388 (6)
Te1—C111	2.150 (4)	C213—H213	0.9500
Te1—C131	2.156 (4)	C214—C215	1.369 (6)
Te1—Br1	3.4481 (9)	C214—H214	0.9500
Te1—Br1^i^	3.3941 (9)	C215—C216	1.385 (6)
Te1—Br2	3.4174 (10)	C215—H215	0.9500
Te2—C211	2.120 (4)	C216—H216	0.9500
Te2—C231	2.134 (4)	C221—C226	1.381 (6)
Te2—C221	2.148 (4)	C221—C222	1.387 (6)
Te2—Br1^i^	3.3922 (9)	C222—C223	1.388 (6)
Te2—Br2	3.3527 (9)	C222—H222	0.9500
C111—C112	1.379 (6)	C223—C224	1.381 (7)
C111—C116	1.391 (6)	C223—H223	0.9500
C112—C113	1.392 (6)	C224—C225	1.376 (8)
C112—H112	0.9500	C224—H224	0.9500
C113—C114	1.383 (7)	C225—C226	1.388 (7)
C113—H113	0.9500	C225—H225	0.9500
C114—C115	1.369 (7)	C226—H226	0.9500
C114—H114	0.9500	C231—C232	1.369 (7)
C115—C116	1.386 (6)	C231—C236	1.378 (6)
C115—H115	0.9500	C232—C233	1.380 (7)
C116—H116	0.9500	C232—H232	0.9500
C121—C126	1.386 (6)	C233—C234	1.385 (8)
C121—C122	1.389 (5)	C233—H233	0.9500
C122—C123	1.370 (6)	C234—C235	1.364 (7)
C122—H122	0.9500	C234—H234	0.9500
C123—C124	1.391 (6)	C235—C236	1.390 (6)
C123—H123	0.9500	C235—H235	0.9500
C124—C125	1.373 (6)	C236—H236	0.9500
C124—H124	0.9500	O1A—C10A	1.166 (17)
C125—C126	1.382 (6)	C10A—C11A	1.43 (2)
C125—H125	0.9500	C10A—C12A	1.58 (2)
C126—H126	0.9500	C11A—H11A	0.9800
C131—C132	1.369 (6)	C11A—H11B	0.9800
C131—C136	1.389 (6)	C11A—H11C	0.9800
C132—C133	1.402 (6)	C12A—H12A	0.9800
C132—H132	0.9500	C12A—H12B	0.9800
C133—C134	1.387 (7)	C12A—H12C	0.9800
C133—H133	0.9500	O1B—C10B	1.170 (15)
C134—C135	1.367 (7)	C10B—C11B	1.416 (19)
C134—H134	0.9500	C10B—C12B	1.59 (2)
C135—C136	1.394 (6)	C11B—H11D	0.9800
C135—H135	0.9500	C11B—H11E	0.9800
C136—H136	0.9500	C11B—H11F	0.9800
C211—C212	1.383 (6)	C12B—H12D	0.9800
C211—C216	1.392 (5)	C12B—H12E	0.9800
C212—C213	1.389 (6)	C12B—H12F	0.9800
C212—H212	0.9500		
			
C121—Te1—C111	91.36 (14)	C214—C213—C212	119.7 (4)
C121—Te1—C131	94.59 (14)	C214—C213—H213	120.2
C111—Te1—C131	96.04 (15)	C212—C213—H213	120.2
C211—Te2—C231	93.58 (15)	C215—C214—C213	120.5 (4)
C211—Te2—C221	94.51 (15)	C215—C214—H214	119.8
C231—Te2—C221	94.37 (17)	C213—C214—H214	119.8
C112—C111—C116	121.2 (4)	C214—C215—C216	120.6 (4)
C112—C111—Te1	119.9 (3)	C214—C215—H215	119.7
C116—C111—Te1	118.8 (3)	C216—C215—H215	119.7
C111—C112—C113	118.9 (4)	C215—C216—C211	119.0 (4)
C111—C112—H112	120.5	C215—C216—H216	120.5
C113—C112—H112	120.5	C211—C216—H216	120.5
C114—C113—C112	120.2 (4)	C226—C221—C222	121.0 (4)
C114—C113—H113	119.9	C226—C221—Te2	122.0 (3)
C112—C113—H113	119.9	C222—C221—Te2	116.9 (3)
C115—C114—C113	120.3 (4)	C221—C222—C223	119.3 (4)
C115—C114—H114	119.9	C221—C222—H222	120.4
C113—C114—H114	119.9	C223—C222—H222	120.4
C114—C115—C116	120.7 (5)	C224—C223—C222	120.0 (5)
C114—C115—H115	119.6	C224—C223—H223	120.0
C116—C115—H115	119.6	C222—C223—H223	120.0
C115—C116—C111	118.8 (4)	C225—C224—C223	120.2 (5)
C115—C116—H116	120.6	C225—C224—H224	119.9
C111—C116—H116	120.6	C223—C224—H224	119.9
C126—C121—C122	119.3 (4)	C224—C225—C226	120.7 (4)
C126—C121—Te1	118.7 (3)	C224—C225—H225	119.7
C122—C121—Te1	122.0 (3)	C226—C225—H225	119.7
C123—C122—C121	120.1 (4)	C221—C226—C225	118.9 (5)
C123—C122—H122	120.0	C221—C226—H226	120.6
C121—C122—H122	120.0	C225—C226—H226	120.6
C122—C123—C124	121.1 (4)	C232—C231—C236	120.0 (4)
C122—C123—H123	119.5	C232—C231—Te2	122.4 (3)
C124—C123—H123	119.5	C236—C231—Te2	117.6 (3)
C125—C124—C123	118.4 (4)	C231—C232—C233	119.6 (5)
C125—C124—H124	120.8	C231—C232—H232	120.2
C123—C124—H124	120.8	C233—C232—H232	120.2
C124—C125—C126	121.4 (4)	C232—C233—C234	121.0 (5)
C124—C125—H125	119.3	C232—C233—H233	119.5
C126—C125—H125	119.3	C234—C233—H233	119.5
C125—C126—C121	119.8 (4)	C235—C234—C233	119.0 (4)
C125—C126—H126	120.1	C235—C234—H234	120.5
C121—C126—H126	120.1	C233—C234—H234	120.5
C132—C131—C136	121.7 (4)	C234—C235—C236	120.4 (4)
C132—C131—Te1	115.9 (3)	C234—C235—H235	119.8
C136—C131—Te1	122.4 (3)	C236—C235—H235	119.8
C131—C132—C133	119.5 (4)	C231—C236—C235	120.0 (4)
C131—C132—H132	120.3	C231—C236—H236	120.0
C133—C132—H132	120.3	C235—C236—H236	120.0
C134—C133—C132	119.1 (4)	O1A—C10A—C11A	129 (2)
C134—C133—H133	120.5	O1A—C10A—C12A	113 (2)
C132—C133—H133	120.5	C11A—C10A—C12A	112 (2)
C135—C134—C133	120.7 (4)	O1B—C10B—C11B	133 (2)
C135—C134—H134	119.6	O1B—C10B—C12B	114 (2)
C133—C134—H134	119.6	C11B—C10B—C12B	112.2 (18)
C134—C135—C136	120.8 (4)	C10B—C11B—H11D	109.5
C134—C135—H135	119.6	C10B—C11B—H11E	109.5
C136—C135—H135	119.6	H11D—C11B—H11E	109.5
C131—C136—C135	118.1 (4)	C10B—C11B—H11F	109.5
C131—C136—H136	120.9	H11D—C11B—H11F	109.5
C135—C136—H136	120.9	H11E—C11B—H11F	109.5
C212—C211—C216	120.7 (4)	C10B—C12B—H12D	109.5
C212—C211—Te2	118.1 (3)	C10B—C12B—H12E	109.5
C216—C211—Te2	121.1 (3)	H12D—C12B—H12E	109.5
C211—C212—C213	119.5 (4)	C10B—C12B—H12F	109.5
C211—C212—H212	120.2	H12D—C12B—H12F	109.5
C213—C212—H212	120.2	H12E—C12B—H12F	109.5
			
C121—Te1—C111—C112	−145.2 (3)	C231—Te2—C211—C212	89.3 (3)
C131—Te1—C111—C112	−50.5 (3)	C221—Te2—C211—C212	−176.0 (3)
C121—Te1—C111—C116	36.6 (3)	C231—Te2—C211—C216	−87.0 (3)
C131—Te1—C111—C116	131.3 (3)	C221—Te2—C211—C216	7.6 (3)
C116—C111—C112—C113	−1.0 (6)	C216—C211—C212—C213	0.9 (6)
Te1—C111—C112—C113	−179.1 (3)	Te2—C211—C212—C213	−175.5 (3)
C111—C112—C113—C114	0.5 (6)	C211—C212—C213—C214	−0.5 (7)
C112—C113—C114—C115	0.1 (7)	C212—C213—C214—C215	−0.8 (7)
C113—C114—C115—C116	−0.4 (7)	C213—C214—C215—C216	1.8 (6)
C114—C115—C116—C111	−0.1 (7)	C214—C215—C216—C211	−1.4 (6)
C112—C111—C116—C115	0.8 (6)	C212—C211—C216—C215	0.0 (6)
Te1—C111—C116—C115	178.9 (3)	Te2—C211—C216—C215	176.3 (3)
C111—Te1—C121—C126	−98.5 (3)	C211—Te2—C221—C226	−89.7 (4)
C131—Te1—C121—C126	165.3 (3)	C231—Te2—C221—C226	4.3 (4)
C111—Te1—C121—C122	78.7 (3)	C211—Te2—C221—C222	91.8 (3)
C131—Te1—C121—C122	−17.5 (4)	C231—Te2—C221—C222	−174.3 (3)
C126—C121—C122—C123	2.4 (7)	C226—C221—C222—C223	0.8 (7)
Te1—C121—C122—C123	−174.8 (3)	Te2—C221—C222—C223	179.4 (4)
C121—C122—C123—C124	0.2 (7)	C221—C222—C223—C224	0.4 (7)
C122—C123—C124—C125	−2.3 (7)	C222—C223—C224—C225	−0.8 (8)
C123—C124—C125—C126	1.6 (7)	C223—C224—C225—C226	−0.1 (8)
C124—C125—C126—C121	1.0 (7)	C222—C221—C226—C225	−1.6 (7)
C122—C121—C126—C125	−3.0 (7)	Te2—C221—C226—C225	179.9 (3)
Te1—C121—C126—C125	174.3 (4)	C224—C225—C226—C221	1.2 (7)
C121—Te1—C131—C132	−103.6 (3)	C211—Te2—C231—C232	4.8 (6)
C111—Te1—C131—C132	164.5 (3)	C221—Te2—C231—C232	−90.0 (6)
C121—Te1—C131—C136	76.9 (3)	C211—Te2—C231—C236	−172.8 (3)
C111—Te1—C131—C136	−14.9 (3)	C221—Te2—C231—C236	92.4 (4)
C136—C131—C132—C133	−1.2 (6)	C236—C231—C232—C233	−1.8 (11)
Te1—C131—C132—C133	179.3 (3)	Te2—C231—C232—C233	−179.4 (7)
C131—C132—C133—C134	1.2 (6)	C231—C232—C233—C234	−0.3 (14)
C132—C133—C134—C135	−0.3 (7)	C232—C233—C234—C235	1.4 (13)
C133—C134—C135—C136	−0.6 (7)	C233—C234—C235—C236	−0.3 (9)
C132—C131—C136—C135	0.3 (6)	C232—C231—C236—C235	2.9 (8)
Te1—C131—C136—C135	179.7 (3)	Te2—C231—C236—C235	−179.4 (3)
C134—C135—C136—C131	0.6 (6)	C234—C235—C236—C231	−1.8 (7)

Symmetry code: (i) −*x*, −*y*, −*z*+1.
